# Heavy Metals in California Women Living in a Gold Mining-Impacted Community

**DOI:** 10.3390/ijerph16132252

**Published:** 2019-06-26

**Authors:** Julie Von Behren, Ruiling Liu, Jane Sellen, Christine N. Duffy, Ryszard Gajek, Key-Young Choe, Josephine DeGuzman, M. Katy Janes, Joanne Hild, Peggy Reynolds

**Affiliations:** 1 Cancer Prevention Institute of California, Fremont, CA 94538, USA; 2 Department of Epidemiology and Biostatistics, University of California San Francisco, San Francisco, CA 94158, USA; 3 Sierra Streams Institute, Nevada City, CA 95959, USA; 4 Environmental Health Laboratory, California Department of Public Health, Richmond, CA 94804, USA

**Keywords:** arsenic, cadmium, heavy metals, gold mining, California

## Abstract

Gold mining activities occurred throughout the foothills of the Sierra Nevada Mountains in California, leaving behind persistent toxic contaminants in the soil, dust, and water that include arsenic and cadmium. Despite a high level of concern among local residents about potential exposure and high breast cancer rates, no biomonitoring data has been collected to evaluate the levels of heavy metals. We conducted a study to characterize the urinary levels of heavy metals among women in this region by working with the community in Nevada County. Sixty women provided urine samples and completed a questionnaire. We examined levels of arsenic, cadmium, and other metals in relation to the length of residency in the area, age, dietary factors, recreational activities, and smoking. We compared urinary metal levels in participants to levels in the United States National Health and Nutrition Examination Survey (NHANES). Overall, study participants had higher urinary levels of arsenic than women in the national sample. Cadmium levels were similar to the national average, although they were elevated in women ≥35 years who had lived in the region for 10 years or more. Arsenic levels were higher among women who smoked, ate fish, ate home-grown produce, and who reported frequent hiking or trail running, although these differences were not statistically significant. This study established a successful community–research partnership, which facilitated community dialogue about possible human health consequences of living in a mining-impacted area.

## 1. Introduction

**Figure 1:**
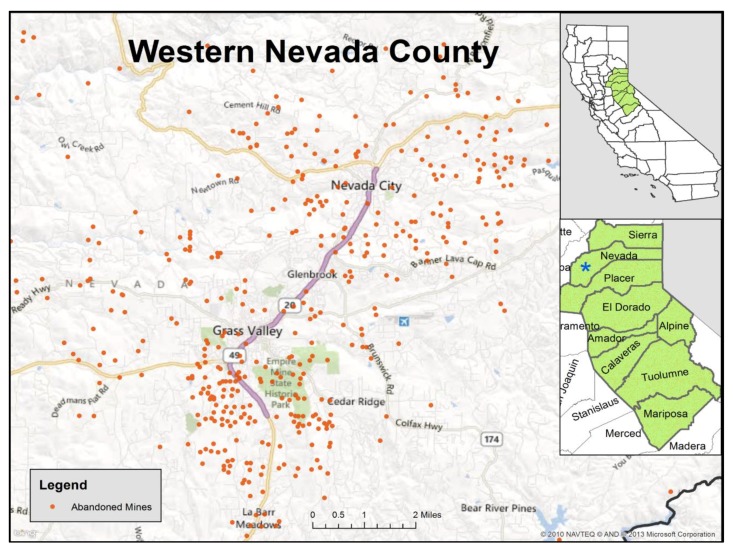
Map of abandoned mine sites in western Nevada County, California. Insets show Gold Country counties. (Source: US Geological Survey).

The foothill region of the Sierra Nevada Mountains in Northern California is impacted by extensive environmental metal contamination as a result of gold mining activities that began with the 1849 Gold Rush (Figure 1). Following the discovery of gold in California in 1848, over 3634 US tons of gold were extracted from the Sierra Nevada foothills using placer, hydraulic, and hard rock mining techniques [1]. The most productive mines were located in Nevada County [2]. As a toxic byproduct of this mining activity, metals such as arsenic (As) and cadmium (Cd), which naturally occur in gold deposits, as well as imported mercury (Hg), used as an amalgam, were released into the environment and are persistent contaminants of soil, surface water, and groundwater in the region. Residents are concerned about potential exposure to these and other heavy metals around abandoned mine sites in their community and near their homes. However, no biomonitoring data have been collected to evaluate the levels of heavy metals in residents of this area.

Arsenic and cadmium are classified as known human carcinogens [3]. Arsenic has been associated with cancers of the bladder, skin, lung, and kidney [4]. Cadmium has also been associated with lung cancer, with suggested links to kidney and prostate cancer [4]. Residents in this historic gold mining area are concerned about the possible breast cancer risk from these environmental contaminations because of the notably high rates observed. In the last ten years of available cancer registry data, 2006–2015, Nevada County had the third highest age adjusted rate of breast cancer in California, while neighboring Placer County had the second highest rate (138 and 142 cases per 100,000 population, respectively, compared to the state’s average of 121 per 100,000) [5]. While links between heavy metals and breast cancer are not well established, toxicological evidence suggests that arsenic and cadmium may act as endocrine disruptors by mimicking estrogen and targeting estrogen receptor-α [6,7,8,9,10].

Because of community concerns about high breast cancer rates, we initiated a study known as CHIME (Community Health Impacts of Mining Exposure) to work with the local community in Nevada County to characterize urinary levels of heavy metals among women in this region. We also wanted to explore behaviors that may be related to increased exposure and engage residents in a dialogue about environmental health issues. With a particular focus on arsenic and cadmium, we examined the levels of metals in relation to length of residency in the area, dietary factors, recreational activities, and smoking history.

## 2. Materials and Methods

This project was conducted as a community/academic partnership between the Sierra Streams Institute (SSI), the Cancer Prevention Institute of California (CPIC), and the University of California, San Francisco (UCSF). SSI, a local watershed monitoring, research, and restoration group based in the Sierra Nevada foothills (Nevada City, CA, USA), provided the community participation for the project. CPIC, a non-profit cancer research organization, and UCSF provided the scientific direction for the study. This study was initially reviewed and approved by the Institutional Review Board of the Cancer Prevention Institute of California, Fremont, CA in December 2013 (IRB Number 2013-008). The project was subsequently reviewed by the Institutional Review Board of the University of California, San Francisco in December 2018 (IRB Number 18-26198). 

### 2.1. Study Population

We recruited 60 women residing in the Nevada County area. The recruitment goal was to enroll a balanced sample of younger and older women, as well as longer- and shorter-term residents of the region. Therefore, roughly half of the participants were under the age of 35 years and half were ≥35 years. Half of each age group had resided in the region for <10 years and half had resided for ≥10 years. Recruitment strategies included advertising through SSI’s monthly e-newsletter and Facebook page, the local newspaper, flyers posted throughout the community in various locations (e.g., the local community college), on the local radio stations, and by word-of-mouth. Interested individuals were then sent a consent form, a brief questionnaire, and a urine collection kit with instructions. First morning urine samples were collected, kept cool while transported, refrigerated at SSI, and then shipped via Fed Ex using a standardized protocol developed between the laboratory and CPIC study manager. Urine samples were received by the lab within 3 days of collection. Signed consent forms and completed questionnaires were returned to CPIC for data entry and analysis. The questionnaire data and samples were collected in the summer of 2014.

### 2.2. Questionnaire Data and Analysis

The self-administered questionnaire was developed in collaboration with, and with input from, the study’s Community Advisory Board (CAB) to focus on factors which might be related to exposure to mining residue. Topics related to residential history, neighborhood characteristics and outdoor recreational activities were covered, with some brief questions about usual diet. We asked for a complete residential history including how long the participant had lived in Gold Country (Figure 1), defined as the nine counties in northeastern California where historic gold mining was concentrated (Placer, El Dorado, Nevada, Tuolumne, Calaveras, Amador, Mariposa, Sierra, and Alpine). The women were asked whether they currently lived on a dirt road, used well water, or had pets in the home. Participants provided their smoking history and answered dietary questions about the consumption of fish, seafood, and locally home-grown produce. They were asked about outdoor activities including hiking, trail running, and gardening. The associations between survey question responses and urinary levels of metals were estimated by comparing geometric means by different responses using the Kruskal–Wallis test for analysis of variance (ANOVA), with values of *p* < 0.05 being statistically significant. To test for potential interactions between age category and length of residency, we used generalized linear models, using the log-transformed values with the link function identify and the normal distribution. The statistical analyses were performed in SAS, version 9.4 (Cary, NC, USA).

### 2.3. Specimen Collection

Participants provided a first morning void urine sample. The urine samples were analyzed for a panel of heavy metals: arsenic, cadmium, mercury, cobalt, manganese, molybdenum, selenium, thallium, tungsten, and uranium. All of these metals are included in the California Biomonitoring Designated Chemicals list. These analyses were performed by the California Department of Public Health, Environmental Health Laboratory in Richmond, California.

### 2.4. Laboratory Methods and Analysis

For the urine analysis, an Agilent 7500 quadrupole ICP-MS (Agilent, Santa Clara, CA, USA)with an octopole collision cell was used. The isotopes and integration times selected are listed in Table 1. Helium was used as a collision gas with a flow rate of 4.5 mL/min to eliminate or reduce a variety of polyatomic interferences affecting accuracy during ICP-MS analysis. The intermediate calibration standards were prepared in a solution of 2% (v/v) nitric acid and 1% (v/v) sulfuric acid. Sodium chloride (1% w/v) and gold (1 mg/L) were introduced as a matrix matching component and a Hg stabilizer, respectively. The sample diluent consisted of 2% (v/v) nitric acid, 2.5% (v/v) 200 proof ethanol, 1 mg/L gold, and 10 to 50 µg/L germanium, rhodium, and rhenium as internal standards. A urine specimen or an intermediate calibration standard was diluted 1:10 with the sample diluent and then delivered to ICP-MS with an integrated flow-injection system. All urine samples were prepared and analyzed in duplicate; the average values of the duplicate measurements were reported. Relative percent differences were typically less than 10% when elemental levels were greater than 10 times the method detection limits. The accuracy of the analytical method was validated by participating in proficiency testing programs, led tri-annually by the Institute National de Santé Publique Québec and the New York State Department of Health. Performance of the instrument was checked on a daily basis by analyzing three levels of internal quality control materials that were prepared with pooled human urine specimens spiked with the analytes of interest. Long-term precision, measured as relative standard deviation, was typically less than 5% for all the analytes over a period of 50 days. The method detection limits (MDLs) were estimated by analyzing seven replicates of a human urine specimen with low levels of the analytes of interest and multiplying the standard deviation of these measurements by a factor of 3.143 (Table 1).

For statistical purposes, values that were below the limit of detection (LOD) were set to 1/√2 LOD. The cadmium values were adjusted for creatinine in urine. Creatinine was analyzed using a BioAssay Systems QuantiChrom^TM^ Creatinine Assay Kit (BioAssay Sytems, Hayward, CA, USA) and a BioTek ELx800 Absorbance Microplate Reader. Urine samples were mixed with a working reagent consisting of a 1:1:1 ratio of deionized water and two reagents (Reagent A and Reagent B) from the assay kit. Reagent A consisted of 1.60% sodium hydroxide and <0.05% ethylenediaminetetraacetic acid (EDTA), and Reagent B consisted of <0.50% picric acid, 20.00% dimethylsulfoxide and <0.20% polyoxyethylenesorbitan monolaurate. The urine-working reagent mixture was incubated at room temperature for approximately 45 min, and the absorbance of the colored adduct was measured at 490 nm to calculated urine creatinine concentrations. 

### 2.5. Arsenic Speciation

For women with total arsenic values ≥20 µg/L (*n* = 12), their samples were speciated for organic and inorganic arsenic by a contract laboratory using the U.S. Environmental Protection Agency EPA Method 1632 (Brooks Applied Labs in Bothell, WA, USA) [11]. Inorganic arsenic, which is more toxic to humans than organic arsenic, was estimated as the total of four chemicals: dimethylarsinic acid (DMA), monomethylarsonic acid (MMA), arsenic acid, and arsenous acid. These are the major metabolites and individually measurable species that result from inorganic arsenic exposure [12,13]. An additional follow-up questionnaire was conducted by telephone and a second urine sample was collected with 6 of the 12 women who had an inorganic arsenic level of ≥20 µg/L (the level of concern identified for follow up by California’s Biomonitoring Program). That questionnaire included detailed questions on potential factors that might be associated with high arsenic levels in urine: consumption of rice, rice-based products, seafood, alcohol, seaweed, and herbal medicines, exposure to pressure-treated wood, and occupational tasks.

### 2.6. Comparison to National Data

We compared the body burden levels of the heavy metals in the CHIME participants to data from the National Health and Nutrition Examination Survey (NHANES), which is designed to be a representative sample of the US population [14]. We compared the levels of metals in the urine of CHIME participants to levels in women ages 22–79 in the NHANES for survey years 2013–2014 (population-weighted). CHIME and NHANES values were compared using the z-test for differences between the means of two groups.

## 3. Results

The women recruited for the study were mostly white non-Hispanic (93%, Table 2), which reflects the community’s demographics. By design, about half of the participants were under age 35 years and about half were ≥35 years. The ages of the participants ranged from 21 to 80 years, and the median age was 36 years. Similarly, by design, participants were equally divided by length of residency in Gold Country (<10 years and ≥10 years). The subjects were generally well-educated with 60% having completed college. Only seven women (12%) were current smokers, and 17 (28%) were former smokers.

**Figure 2:**
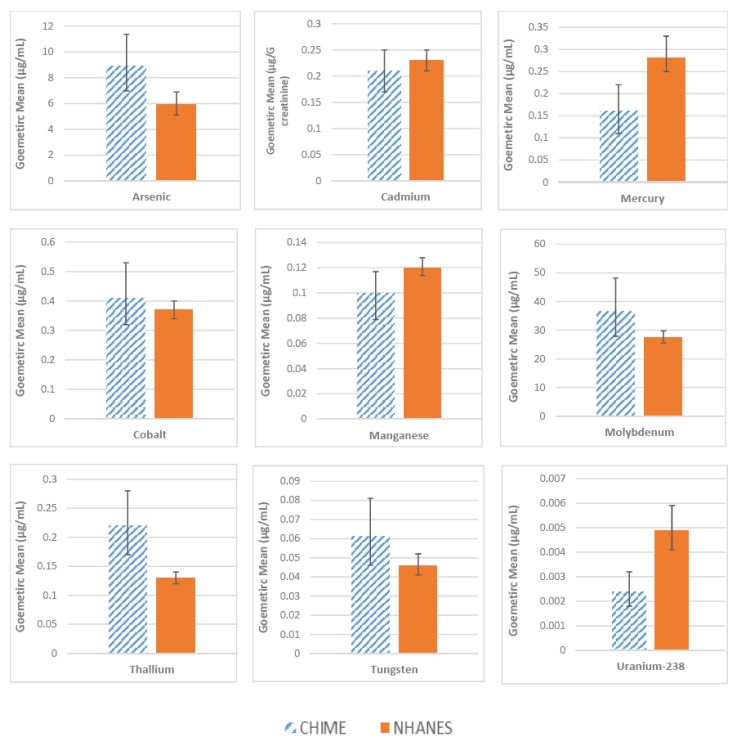
Metal levels in urine among study participants (CHIME), Nevada Country, California, compared to data from the National Health and Nutrition Examination Survey (NHANES) with 95% confidence bars.

The levels of the metals measured in urine are shown in Figure 2. We compared the geometric mean values in CHIME to those of similarly-aged women in the NHANES. The mean total arsenic level in the CHIME study participants was statistically significantly higher than for women in the national sample (8.81 and 5.94 µg/L, respectively). Cadmium levels in the CHIME women were similar to the national sample average (0.21 µg/g creatinine compared to 0.23 µg/g creatinine). We observed higher levels of molybdenum and thallium among the participants, whereas the levels of mercury, manganese, and uranium were lower in the CHIME women as compared to the NHANES data. The levels of cobalt and tungsten were not statistically different in the two groups. The mean urinary selenium level in the study sample was 42.82 µg/L (95% Confidence Interval 35.96, 51.01). Urinary selenium was not measured in NHANES.

A total of 12 women had arsenic levels above 20 µg/L, the level of concern. Their arsenic levels were speciated to determine inorganic arsenic levels. Of those 12 participants, only six had total inorganic arsenic levels ≥20 µg/L. An additional questionnaire and first morning urine sample were collected from these participants. The responses to the questionnaire, which included questions on the consumption of food items potentially high in arsenic, including rice, rice-based products, seafood, alcohol, seaweed, and herbal medicines. The frequency distributions for this small sample of six participants did not reveal any clear patterns. Upon testing of the second urine sample from the six women with elevated inorganic arsenic values, none exceeded the level of concern, 20 µg/L.

The measured levels of urinary arsenic and cadmium by age, smoking status, household characteristics, diet, and activities are shown in Table 3. Arsenic levels were higher in women under the age of 35 years (11.48 µg/L) than in women aged ≥35 years (7.24 µg/L, *p* = 0.06). Arsenic levels were elevated among women with less than 10 years of residency compared to those with ≥10 years of residency (10.23 vs. 7.24 µg/L), although this difference was not statistically significant. Arsenic levels were also higher among women who were current smokers and those who reported frequent and recent fish consumption, frequent consumption of local home-grown produce, and frequent hiking/trail running, although these differences were not statistically significant. Cadmium levels were higher in women aged ≥35 years than in women aged less than 35 years (0.28 µg/g creatinine and 0.14 µg/g creatinine respectively, *p* < 0.05). Cadmium levels were modestly elevated among current smokers, women who reported currently living on a dirt road, having frequent and recent fish consumption, having a dog or cat that spends time outside, and frequently gardening, although again these differences were not statistically significant. Cadmium levels were lower in women who reported frequent hiking and trail running than those who did not report hiking or running (0.16 µg/g creatinine and 0.30 µg/g creatinine respectively, *p* < 0.05).

We examined the levels of metals by both age category and length of residency (Table 4). Although cadmium levels in CHIME participants were lower than the national sample, they were significantly elevated in women aged ≥35 years old who had lived in Gold Country ≥10 years (Table 4, *p* = 0.006 for age and residency interaction). For arsenic, the opposite pattern emerged, with levels lower among older women with ≥10 years of residency in the area (*p* value = 0.23 for age and residency interaction).

## 4. Discussion

In this mining-impacted community, study participants had higher urinary levels of arsenic than adult women in the national sample. Cadmium levels were similar to the national sample average. Older women with longer length of residency in the area had higher levels of cadmium. Because of the accumulative properties of cadmium, levels were elevated with increasing age. Cadmium accumulates in the body, and urinary cadmium is a marker of long-term exposure. Urinary arsenic, however, is a marker of recent exposures. The half-lives of arsenic and arsenic metabolites in urine range from a few hours to a few days [15,16], whereas the half-life of Cd in urine is more than 13 years [17,18].

Because of the presence of metals in the dust, soil, and water in this region of California, exposure can occur from common activities such as outdoor gardening, hiking, running, or walking on dirt trails. About 55% of the women in this study reported weekly hiking or trail running, and 68% reported gardening once a month or more. Ingestion exposure may occur through the consumption of fruits and vegetables grown in contaminated soil. These contaminants can accumulate in commonly grown garden plants like broccoli, kale, lettuce, and potatoes [19,20,21,22]. Seventy-five percent of the women in the study reported weekly consumption of locally home-grown produce. In this small study sample, we observed that women who reported consuming locally home-grown produce once a week or more had higher arsenic levels than those who did not (10.23 µg/L vs. 6.03 µg/L respectively), though this difference was not statistically significant. Conversely, levels of cadmium were lower in the group that consumed more garden produce (0.19 µg/g creatinine vs. 0.24 µg/g creatinine respectively). These differences could be due to chance. It is also possible that arsenic may be more bioavailable than cadmium through the ingestion of plants [23].

The ability of cadmium and arsenic to bind to and activate the estrogen receptor suggests that exposure to these metals may contribute to the etiology of breast cancer [6,7,8,9,10]. This hypothesis is supported by a handful of epidemiologic studies that have observed associations between increased breast cancer risk and cadmium exposure [24,25,26]. However, a recent Danish prospective study found no association between urinary cadmium levels and postmenopausal breast cancer risk [27]. The evidence for arsenic and breast cancer risk is even sparser and more mixed [28,29].

Since the first year of statewide reporting of all newly diagnosed cancers in California in 1988, breast cancer incidence rates in Gold Country have been consistently higher than the state average [5]. According to the most recent ten years of data from the California Cancer Registry (2006–2015), several counties in Gold Country had age-adjusted annual incidence rates for invasive breast cancer that exceeded the statewide rate of 121.4 cases per 100,000 women. The three most populous counties in Gold Country had breast cancer incidence rates that were similar to the highly publicized elevated rate in Marin County (138.2 in Nevada County, 131.7 in El Dorado County, 142.0 in Placer County, and 142.9 in Marin County). While it is not known whether this high breast cancer incidence may be, in part, attributable to environmental contamination unique to Gold Country, continued investigation of the effects of exposure to these metals on the risk of breast cancers is warranted given the carcinogenic and endocrine disrupting properties of cadmium and arsenic that pollute this region, along with the potential for exposure and the high level of concern among residents.

Among the additional metals included in this study, cobalt is also potentially carcinogenic according to the National Toxicology Program 14th Report on Carcinogens and the International Agency for Research on Cancer. There are other possible health effects from the metals included in this study, including harm to the nervous system from mercury and thallium. Also, there is little knowledge about the potential synergistic effects that may result from exposure to multiple metals. The levels of these additional metals could be used in the future as reference values.

This study had some notable limitations, primarily the small sample size of 60 women. This small sample limited our ability to address potential confounding factors through more detailed multivariable analyses. In addition, the study was based on a convenience sample of women representing certain age and residency criteria. It is important to note that arsenic in urine is a marker of short-term exposure, not long term, which would be more important for assessing potential cancer risk [13]. We did not collect any environmental samples or conduct any exposure assessment. Furthermore, all of the participants’ activities and behaviors were self-reported.

## 5. Conclusions

This study established a successful community–research partnership, which facilitated community dialogue about the possible human health consequences of living in a mining-impacted area. The measured levels of arsenic were higher in this study than in the national comparison group, and cadmium levels were higher in older, long-term residents. Community residents remain concerned about possible exposures to heavy metals, especially through common activities such as gardening, eating home-grown produce, hiking, and trail running. To better understand the exposure potential identified from self-reported behaviors, additional follow-up studies are currently underway in Gold Country. These studies include recruiting additional participants and training them to collect household samples of dust, soil, and water, as well as testing vegetables grown in school gardens for arsenic and cadmium.

## Figures and Tables

**Table 1:** Isotopes monitored, method detection limits (MDLs), and number of samples below MDL for the metals of interest.

Analyte	Isotope	Integration Time (s)	MDL (µg/L)	# Samples out of 60 below MDL
Arsenic	^75^As	1.5	0.0493	0
Cadmium	^111^Cd	2.0	0.0420	3
Mercury	^202^Hg	2.0	0.0163	3
Cobalt	^59^Co	1.0	0.0113	0
Manganese	^55^Mn	1.0	0.0499	13
Molybdenum	^95^Mo	1.0	0.0736	0
Selenium	^78^Se	2.0	0.343	0
Thallium	^205^Tl	1.0	0.00982	0
Tungsten	^182^W	1.0	0.00493	3
Uranium	^238^U	1.0	0.00134	27

**Table 2:** Demographic characteristics of the 60 female study participants, 2014, Nevada County, California.

Characteristic	Study Subjects *N* = 60
Age	range 21–80 years
<35 years	27 (45%)
≥35 years	33 (55%)
Race/ethnicity	
Non-Hispanic white	56 (93%)
Other	4 (7%)
Educational level	
High school graduate or less	4 (7%)
Some college or trade school	20 (33%)
College graduate	36 (60%)
Length of residency in Gold Country	
<10 years	30 (50%)
≥10 years	30 (50%)
Smoking status	
Current smoker	7 (12%)
Former smoker	17 (28%)
Never smoker	36 (60%)

**Table 3:** Urinary arsenic and cadmium levels versus answers to survey questions on residential features, diet, and activities.

Survey Question	Number of Participants	Arsenic (µg/L)	Cadmium (µg/g creatinine)
		Geometric Mean	Geometric Mean
Age			
<35 years	27	11.48	0.14
≥35 years	33	7.24 *	0.28 **
Length of residency in Gold Country			
<10 years	30	10.23	0.19
≥10 years	30	7.76	0.22
Smoking status			
current	7	15.14	0.24
former	17	9.12	0.20
never	36	7.94	0.20
Current residence on dirt road?			
yes	18	7.59	0.25
no	42	9.55	0.19
Well at current residence?			
yes	22	8.13	0.18
no	38	9.33	0.22
Outside dog or cat at current residence?			
yes	43	8.91	0.21
no	17	8.71	0.19
Frequency of seafood consumption in past year?			
never	8	5.75	0.17
once a month or less	41	8.51	0.20
once a week or more	11	14.1	0.24
Eaten fish or seafood in last 72 h?			
yes	7	12.30	0.30
no	52	8.32	0.20
Frequency of home-grown produce consumption in past year?			
once a month or less	15	6.03	0.24
once a week or more	45	10.23	0.19
How often did you garden in the past year?			
never or rarely	19	10.96	0.18
once a month	8	5.13	0.20
once a week or more	33	8.91	0.22
How often did you hike or trail run in the past year?			
never or rarely	16	6.03	0.30
once a month	11	9.12	0.26
once a week or more	33	10.72	0.16 **

Unknown or missing responses omitted; * *p* = 0.06; ** *p* < 0.05.

**Table 4:** Geometric Mean Arsenic and Cadmium levels by length of residence in Gold Country and age group.

Metal	Age Group	Length of Residency in Gold Country	
		<10 years	≥10 years
Arsenic	<35 years	11.28	11.63
	≥35 years	9.56	5.60
Cadmium	<35 years	0.17	0.12
	≥35 years	0.21	0.37 **

Results are reported as micrograms per liter (µg/L) for arsenic and micrograms per gram creatinine for cadmium. ** *p* = 0.006.
